# Genetic mapping and QTL analysis for peanut smut resistance

**DOI:** 10.1186/s12870-021-03023-4

**Published:** 2021-07-02

**Authors:** Francisco J. de Blas, Cecilia I. Bruno, Renee S. Arias, Carolina Ballén-Taborda, Eva Mamani, Claudio Oddino, Melina Rosso, Beatriz P. Costero, Marina Bressano, Juan H. Soave, Sara J. Soave, Mario I. Buteler, J. Guillermo Seijo, Alicia N. Massa

**Affiliations:** 1 Instituto Multidisciplinario de Biología Vegetal Consejo Nacional de Investigaciones en Ciencia y Tecnología (CONICET) y Universidad Nacional de Córdoba (UNC), Av. Vélez Sarsfield 1666, X5016GCN Córdoba, Argentina; 2 Genética, Facultad de Ciencias Agropecuarias – UNC, Av. Ing. Agr. Félix A. Marrone 735, CP5001, Córdoba, Argentina; 3 Estadística y Biometría, FCA – UNC, Córdoba, Argentina; 4 CONICET, Av. Ing. Agr. Félix A. Marrone 735, CP5001, Córdoba, Argentina; 5 USDA-ARS-National Peanut Research Laboratory (NPRL), Dawson, GA 39842 USA; 6 Center for Applied Genetic Technologies and Institute of Plant Breeding, Genetics and Genomics, University of Georgia, Athens, GA USA; 7 Instituto Nacional Tecnología Agropecuaria (INTA), Ruta Nac. nro. 9 km 636 Estación Experimental Agropecuaria Manfredi, EEA, X5988 Manfredi, Córdoba, Argentina; 8 Facultad de Agronomía y Veterinaria, Universidad Nacional de Río Cuarto (FAV-UNRC), Ruta Nacional 36, X5804BYA Córdoba, Argentina; 9 Criadero El Carmen, Bv. Italia 835, CP5809, Gral. Cabrera, Córdoba, Argentina; 10 Biología Celular, FCA - UNC, Av. Ing. Agr. Félix A. Marrone 735, CP5001, Córdoba, Argentina; 11 Instituto de Botánica del Nordeste (CONICET-UNNE) and Facultad de Ciencias Exactas y Naturales y Agrimensura, Universidad Nacional del Nordeste, Corrientes, Argentina

**Keywords:** *Arachis*, Disease resistance, Introgression, Peanut, Groundnut, QTL, Peanut smut, RIL population

## Abstract

**Background:**

Peanut smut is a disease caused by the fungus *Thecaphora frezii* Carranza & Lindquist to which most commercial cultivars in South America are highly susceptible. It is responsible for severely decreased yield and no effective chemical treatment is available to date. However, smut resistance has been identified in wild *Arachis* species and further transferred to peanut elite cultivars. To identify the genome regions conferring smut resistance within a tetraploid genetic background, this study evaluated a RIL population {susceptible *Arachis hypogaea subsp. hypogaea* (JS17304-7-B) × resistant synthetic amphidiploid (JS1806) [*A. correntina* (K 11905) × *A. cardenasii* (KSSc 36015)] × *A. batizocoi* (K 9484)^4×^} segregating for the trait.

**Results:**

A SNP based genetic map arranged into 21 linkage groups belonging to the 20 peanut chromosomes was constructed with 1819 markers, spanning a genetic distance of 2531.81 cM. Two consistent quantitative trait loci (QTLs) were identified *qSmIA08* and *qSmIA02/B02*, located on chromosome A08 and A02/B02, respectively. The QTL *qSmIA08* at 15.20 cM/5.03 Mbp explained 17.53% of the phenotypic variance, while *qSmIA02/B02* at 4.0 cM/3.56 Mbp explained 9.06% of the phenotypic variance. The combined genotypic effects of both QTLs reduced smut incidence by 57% and were stable over the 3 years of evaluation. The genome regions containing the QTLs are rich in genes encoding proteins involved in plant defense, providing new insights into the genetic architecture of peanut smut resistance.

**Conclusions:**

A major QTL and a minor QTL identified in this study provide new insights into the genetic architecture of peanut smut resistance that may aid in breeding new varieties resistant to peanut smut.

**Supplementary Information:**

The online version contains supplementary material available at 10.1186/s12870-021-03023-4.

## Background

The cultivated peanut (*Arachis hypogaea* L.) is an important allotetraploid (AABB) originated in South America [1–3]. This legume crop is grown in more than 100 countries with a global production of 47.10 million tones [4]. It is a row crop rich in oil, protein, vitamins and other micronutrients [5], consumed worldwide as nuts, candy, peanut butter and oil. In the last decade, peanut production and industrialization has been threatened in Argentina by the emergence of a new soilborne disease, the peanut smut, caused by *Thecaphora frezii*. This disease causes hypertrophy of pod tissues and seeds colonized with teliospores have a smutted mass appearance [6–8]. Disease incidence may reach up to 52% in commercial plots, with up to 35% yield losses [9, 10]. Cultural management strategies and chemical treatment have not been effective and currently the prevalence of the disease in the peanut production area of Argentina has reached 100% [11].

The fungus *T. frezii* was originally described in the wild *A. kuhlmanii* Krapov. & W.C. Gregory [12] from Aquidauana, Mato Grosso, Brazil [13], and more recently identified in other wild species from Bolivia, such as *A. kempff-mercadoi* Krapov., W.C. Greg. & C.E. Simpson [14]. The initial classification of the fungus, which was based on disease symptoms and morphology of teliospores, was recently confirmed using molecular tools such as the 28S rDNA sequence [15] and its mitogenome sequence [16]. Because seeds of wild species collected in Bolivia, Brazil and Argentina were delivered to main germplasm banks of the world, peanut smut constitutes a threat to the peanut industry around the world.

While moderate results with high doses of fungicide for *T. frezii* have been reported recently [17], the development of smut-resistant cultivars appears as the most efficient, sustainable, and environmentally friendly approach to control smut disease in peanut production. Natural sources of resistance to smut found in some old peanut landraces and in wild relatives can be utilized to enhance peanut performance under high disease pressure [18]. The introgression of smut resistance into elite peanut lines and pre-breeding materials from landraces [19, 20] and wild *Arachis* species [21] were recently reported by our research group. In Bressano et al. [19], the recombinant inbred lines (RILs) involved crosses between three susceptible peanut elite cultivars (*Arachis hypogaea L. subsp*. *hypogaea*) and two resistant landraces (*Arachis hypogaea L. subsp*. *fastigiata* Waldron). While in de Blas et al. [21], a RIL population was derived from a cross between a synthetic amphidiploid (AAKK) and a susceptible experimental line of *A. hypogaea*. Both studies showed significant levels of phenotypic variation and high broad sense heritability for the trait, suggesting that smut resistance is controlled by few major genetic loci. However, the genetic structure of the character is still unknown, and quantitative trait loci (QTLs) have not been identified.

Conventional breeding of new resistant varieties faces great challenges for those characters in which the genetic structure is unknown [22]. Under such circumstances, it is essential to identify QTLs and linked markers for smut resistance, which could be deployed in genomics-assisted breeding (GAB) to accelerate the breeding process [23, 24]. Molecular breeding has already demonstrated its strength in accelerated improvement of target traits in peanut including disease resistance traits [24]. The availability of reference genomes in public databases (https://peanutbase.org) for peanut [2] and its diploid progenitors [25, 26], enhance the potential of sequence analysis, annotation, candidate gene discovery, and marker development. Further, populations of recombinant inbred lines are valuable tools for generating multi-year phenotyping data to elucidate environmental effects on target traits.

In this study, the F_7:9_ RIL mapping population derived from a cross between *A. hypogaea* and a synthetic amphidiploid [(*A. correntina* × *A. cardenasii*) × *A. batizocoi*]^4×^ [21] was used to 1) identify genome regions that confer smut resistance within a tetraploid genetic background; and 2) understand the genetic structure of the trait. The construction of the genetic map and the QTL analysis allowed the detection of two genomic regions introgressed from wild *Arachis* species in a tetraploid background, with major control of peanut smut. The genome locations of both QTLs here identified are rich in genes encoding proteins that are involved in plant defense, providing new insights into the understanding of the genetic architecture of peanut smut resistance.

## Results

### Phenotypic variation for smut resistance

**Fig. 1:**
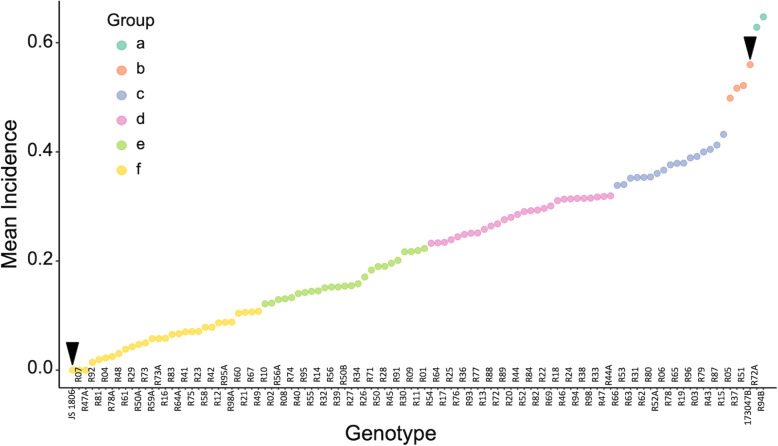
Clusters of recombinant inbred lines (RILs) according to the mean incidence per genotype obtained by Scott and Knott analysis. Clusters are defined as Groups a, b, c, d, e and f. Resistant and susceptible parents are indicated by black arrowheads

Disease incidence in the F_7:9_ RIL population showed statistically significant differences (*P* < 0.01) between years, with average incidence of 0.17, 0.23, and 0.27 for 2016, 2017, and 2018, respectively. The genetic variance component was larger than the genotype × year interaction [56.2% (*P* < 0.01) vs. 5.2% (*P* = 0.0017) for incidence]. Despite statistically significant differences among years, the relative incidence values among lines remained the same. For the susceptible parent, the average incidence of peanut smut was 56% whereas for the amphidiploid, incidence was 0%. The pairwise mean comparison by Scott and Knott test clustered the RILs into six groups (Fig. 1). A cluster of twenty-nine lines had an incidence value < 0.11 and did not show significant differences from the amphidiploid and wild species (resistant control lines). The average incidence of these lines was 0.06 with a range between 0.00 and 0.11. Within the group of resistant lines, six (R04, R07, R47A, R78A, R81, R92) were highly resistant with an average incidence < 0.01. On the opposite extreme of the graph, three lines presented an incidence score > 0.5 and did not show significant differences from the susceptible parental line JS173047-B. Transgressive segregation was observed for two lines (94B and 72A), with incidence significantly higher than the susceptible parent, representing 1.9% of the progeny. Broad-sense heritability (*H*^2^) was statistically significant with a combined value of 0.95.

### Genotyping, genetic mapping, and single marker analysis

The genotyping of the 103 RILs, parental lines, and diploid progenitors with the 48 K ‘Axiom_Arachis2’ SNP array retrieved a total of 7496 polymorphic SNP markers (15.62%), 3662 assigned to the A-subgenome and 3834 to the B/K subgenome. Different numbers of species-specific markers for the diploid progenitors of the amphidiploid were found: 99 SNPs for *A. correntina* (A genome), 98 for *A. cardenasii* (A genome), and 1139 for *A. batizocoi* (K genome). Moreover, a total of 3456 SNPs markers were specific to *A. hypogaea*, 1268 A-subgenome and 2158 B-subgenome specific markers (E-value <1e-20). Most SNPs were inherited as expected for disomic segregation, except for 209 SNPs, which presented a genotype graph display more compatible with tetrasomic inheritance according to [27]. Fifty six percent of the markers showed segregation distortion at *P* < 0.001. They were distributed in few discrete clusters on linkage groups (LGs) A04, A05, A07, B03, and B04 (Additional file 1). These clusters were considered as segregation distortion regions (SDRs) since at least four skewed markers were clustered in each other LG.

**Fig. 2:**
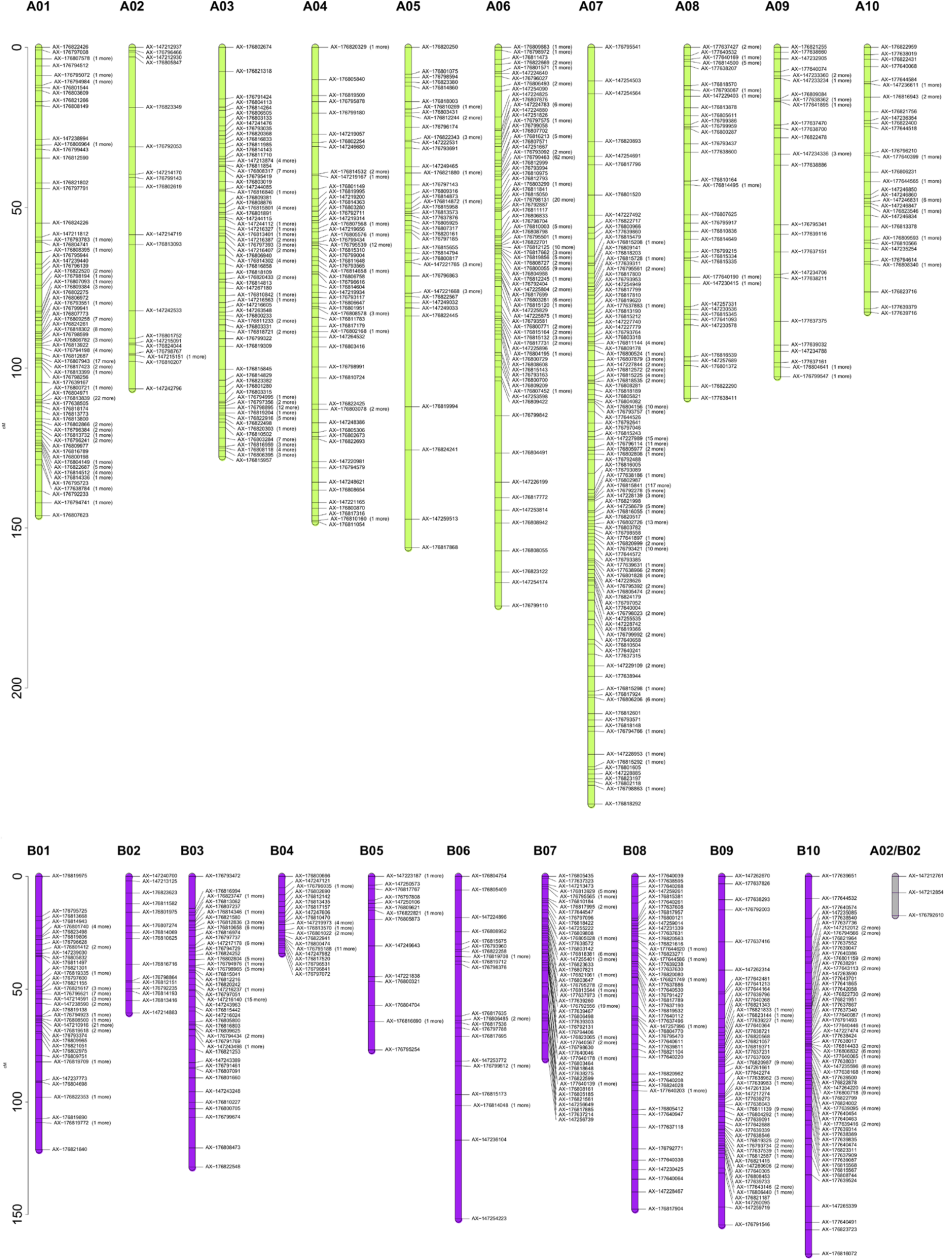
Genetic linkage map constructed for the 103 RIL population derived from the cross of *A. hypogaea* JS17304–7-B × the synthetic amphidiploid JS1806. The 1819 SNP markers are shown at the right side the 21 linkage groups and the number of co-localized markers are indicated in brackets. Each marker is indicated with the probeset name provided by 48 K ‘Axiom_Arachis2’ SNP array [28]. A-subgenome LGs are represented in green, B-subgenome LGs are in purple and LG with no genome assignation is in gray. The bars in the y-axis represent the genetic distance in cM

The linkage map was constructed in two steps. First, a set of 918 frame markers, selected after filtering for non-distorted and non-co-located markers, were utilized. While for the second step, 889 co-located and distorted markers (with up to 4:1 segregation ratio) were forced to integrate into the linkage groups, as long as they did not change the markers original order and mapped distance. The physical position of the A-genome and B-genome markers was defined by BLASTn tool according to the reference sequences of *A. hypogaea* cv. Tifrunner [2] (https://peanutbase.org). The resulting linkage map consisted of 1819 SNP markers assigned to the 20 peanut chromosomes. The mapped SNP markers spanned a total genetic distance of 2531.81 cM (Table 1; Fig. 2). The linkage map was arranged into 21 LGs from which 20 LGs were assigned to a particular subgenome (A or B) and one short LG belonging to the homoeologous group 2, which was kept as an independent fragment with no assignation to either A or B subgenomes. This fragment, named as LG A02/B02, was retained as part of the map as it held two SNPs significantly associated to peanut smut incidence. The size of the LGs varied from 17.28 cM (LG A02/B02) to 236.22 cM (LG A07). The average distance between adjacent markers was of 1.41 cM and ranged from 0.66 cM (LG A07) to 6.08 cM (LG B06). The number of markers mapped on each linkage group ranged from 15 (LG B02) to 357 (LG A07). The names of the linkage groups were assigned based on the assignment of SNPs to the sequence-based pseudomolecules (https://peanutbase.org). The only exception was LG A02/B02, with 3 SNPs and 17.29 cM, which could not be assigned to a particular subgenome. Thus, the QTL was named using both genome designations.

**Table 1:** Statistical data of the genetic map obtained by the analysis of the 103 *Arachis* RIL population

LG	No. of mapped SNPs	Map length	Average distance	Maximum distance
A01	168	146.24	0.88	10.68
A02	20	106.51	5.61	20.70
A03	144	127.67	0.89	8.52
A04	83	148.15	1.81	10.02
A05	54	156.16	2.95	28.25
A06	239	174.40	0.73	11.69
A07	357	236.22	0.66	14.96
A08	49	109.45	2.28	9.93
A09	35	102.70	3.02	18.53
A10	44	82.60	1.92	8.79
B01	87	121.19	1.41	17.57
B02	15	60.51	4.32	11.51
B03	90	129.12	1.45	14.25
B04	39	34.08	0.90	5.13
B05	17	76.96	4.81	13.76
B06	26	151.94	6.08	35.00
B07	95	80.95	0.86	7.01
B08	54	147.56	2.78	9.50
B09	89	154.61	1.76	14.92
B10	111	167.51	1.52	13.91
A02/B02	3	17.29	8.64	10.02
**Total**	**1819**	**2531.81**		

The SNP markers per linkage group (LG) are expressed in absolute numbers. Map length, average spacing between markers, and maximum distance between markers are expressed in cM

After performing a single martker analysis to detect SNPs associated to peanut smut incidence, 236 SNPs were found statistically significant (*P <* 0.01) in LGs A01, A03, A07, A08, A02/B02, B02, B03 and B09 (Additional file 2).

### Evaluation of the genetic map

The markers positioned in the genetic map showed a relatively strong collinearity with the physical position reported by Bertioli et al. [2] for the tetraploid *A. hypogaea* cv. Tifrunner reference genome (https://peanutbase.org). A total of 1819 loci were anchored to the *A. hypogaea* pseudomolecules with an average physical interval of 1.39 Mbp between loci and a total physical distance of 2527.56 Mbp. The average recombination rate was 3.59 cM/Mbp with a maximum rate of 17.16 cM/Mbp (A07). The LGs A10, B03, B04, B05 and B06 had the lowest recombination rates (0.21, 0.9, 0.14, 0.84, 0.01 cM/Mbp, respectively). In most cases the loci were evenly distributed along the chromosomes but in other cases, the analysis showed a higher marker density and increased recombination in the distal portion of the chromosome arms and lower marker density and recombination frequency in the pericentromeric regions. Most of the linkage groups showed good concordance with the pseudomolecules. The only exception was a set of markers located in a segment (76–88 cM) of chromosome A05, which were observed as an arc line relative to the rest of the markers in a linkage group (A05 in Additional file 3). This arc is formed due to the opposite orientation of the markers with respect to the physical map (Additional file 3), evidencing an inversion. The marker-pairwise recombination fractions versus LOD scores (Additional file 4) reflected a strong linkage between each pair of markers within LGs, except for markers that seem to be tightly associated between LGs A03/B03 and LGs A04/B04.

### QTL for peanut smut resistance

**Fig. 3:**
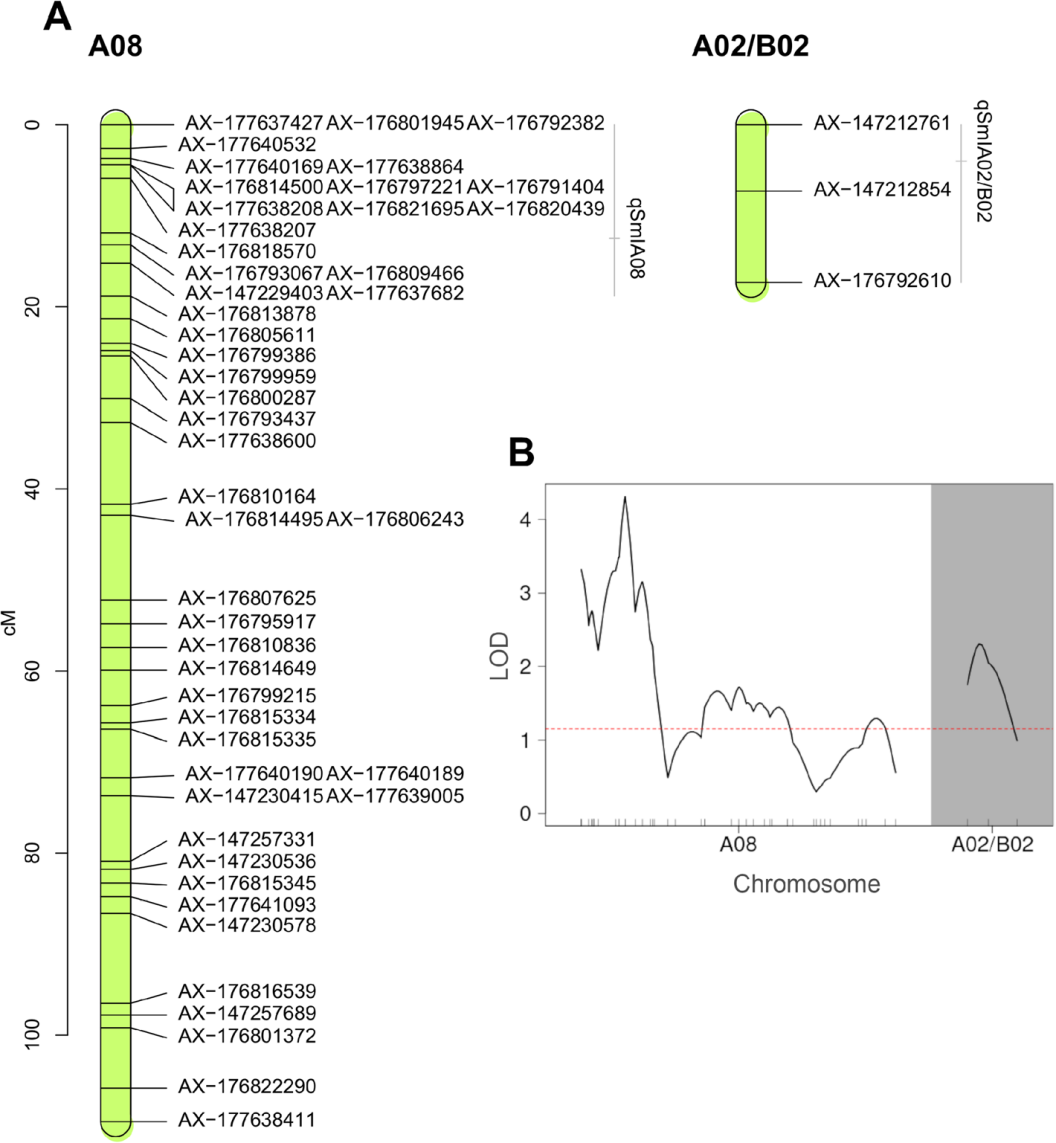
QTLs for peanut smut resistance. A: Detail of the A08 and A02/B02 LGs showing the QTLs between *lodint* intervals as vertical bars and the peak as a horizontal dash. Y-axis indicates the genetic distance. Marker names (probesets 48 K ‘Axiom_Arachis2’ SNP array) are indicated on the right of each LGs. B: LOD scores per LGs A08 and B02; red dashed horizontal line indicates the empirical LOD score threshold at *P*-value < 0.01

**Fig. 4:**
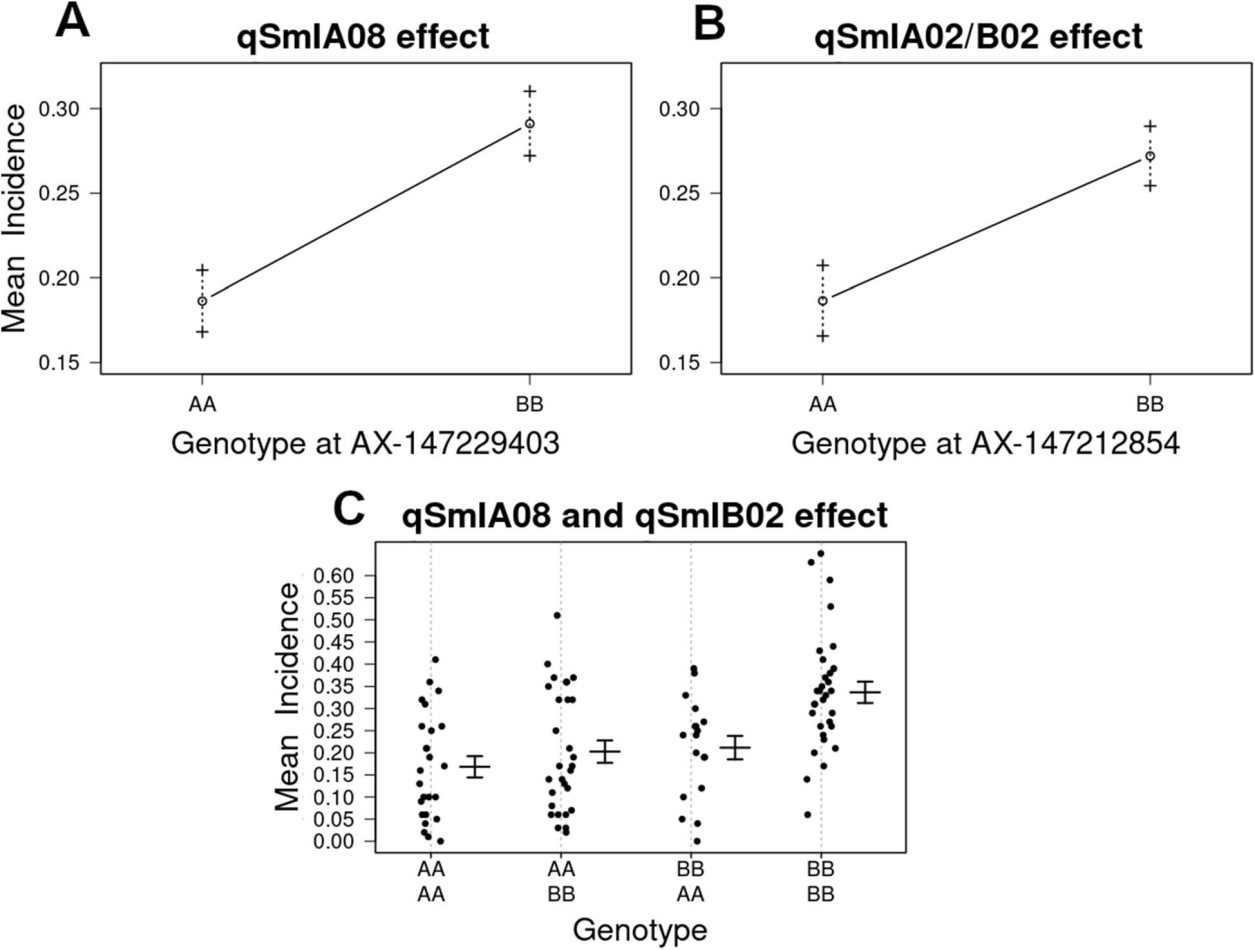
Phenotypic effects of markers tightly linked to QTLs contributing to peanut smut resistance. A: effect of genotype at AX-147229403. B: effect of genotype at AX-147212854. C: Combined effect of genotypes at AX-147229403 and AX-147212854. AA and BB correspond to the amphidiploid and to the peanut elite line genotypes, respectively

The QTL analysis identified SNPs associated to smut disease incidence on chromosomes A01, A07, B02, B03 and B09, with significant LOD scores in at least two of the 3 years of disease evaluation. However, only two chromosome regions (A08, A02/B02) were significantly associated with incidence across the 3 years of field evaluation (Additional file 5; Additional file 6). The approximate physical positions of the QTLs were defined by the closest genetic markers. The QTL *qSmIA08* was detected in LG A08 at 15.20 cM (AX-147229403, 5.03 Mbp) (MAF = 0.5), while the *qSmIA02/B02* was found at four cM from the beginning of the LG A02/B02 (AX-147212854, 3.56 Mbp) (MAF = 0.48). Both QTLs showed LOD scores above the empirical threshold at *P*-value = 0.01 (Table 2; Fig. 3). The one-way ANOVA with a post-hoc TukeyHSD test and Bonferroni’s correction (*P* = 0.05) showed statistically significant differences of peanut smut incidence among groups of genotypes at the AX-147229403 and AX-147212854 positions. The haplotype analysis showed independence of QTLs at these marker positions. The analysis of the phenotypic effects of markers tightly linked to QTLs contributing to peanut smut resistance revealed that most (86%) RILs with amphidiploid alleles showed the lowest phenotypic mean scores, while all RILs carrying *A. hypogaea* alleles had a significant higher incidence. The statistically significant reductions of incidence for individual QTLs and for both QTLs combined are shown in Fig. 4. The additive effect values detected for each QTL (Table 2) suggest that these genome segments contributed to reduce peanut smut incidence by 40.87 and 32.07%, respectively. The analysis of the two QTLs assessed together showed that when RILs carry both alleles from the amphidiploid (that is, *qSmIA08* AA and *qSmIA02/B02* AA) there is a decrease in disease incidence (56.15%, *P* < 0.001) (Fig. 4, Table 3). Furthermore, statistically significant differences (*P* < 0.05) were observed between phenotypes carrying the allele AA at *qSmIA08* and BB at *qSmIA02/B02*, and vice versa.

**Table 2:** Detail of the QTLs detected for resistance to peanut smut on a RIL population of *Arachis* based on a Halley-Knott genome scan QTL detection model

LG^**a**^	Genetic Position^**b**^	Physical Position^**c**^	SNP Marker	LOD^**d**^	LOD threshold^**e**^	95% Bayes interval^**f**^	LOD interval^**g**^	Additive effect^**h**^	PVE^**i**^	% ^**j**^
A08	15.2	5.03	AX-147229403	4.31	2.04	0–71.72	0–18.78	0.12	17.52	40.87
A02/B02	4	3.56	AX-147212854	2.31	1.15	0–17.28	0–17.28	0.105	9.06	32.07

Resistance to peanut smut is estimated for the level of square root transformed smut incidence; ^a^Linkage group. ^b^Genetic position in Kosambi cM for each LG. ^c^Physical position (Mbp) based on *A. hypogaea* cv. Tifrunner pseudomolecules [2] (https://peanutbase.org). ^d^LOD score at QTL peak. ^e^LOD threshold based on 1000 permutations at 1% level of significance. ^f^95% Bayes credible intervals. ^g^LOD support interval. ^h^Additive effect values, positive values indicate that alleles come from one of the wild diploid species (2*n* = 2*x* = 20) (*A. correntina, A. cardenasii* or *A. batizocoi*) and negative values indicate that alleles come from the *A. hypogaea* experimental elite line JS17304-7-B (2*n* = 4*x* = 40). ^i^Proportion of the phenotypic variance explained by the QTL. ^j^Percentage (%) of decrease in peanut smut incidence

**Table 3:** Pairwise comparisons between phenotypes carrying different combination of allele (AA-BB) at *qSmIA08* and *qSmIA02/B02* SNP positions

Pairwise comparison^**a**^	Differences^**b**^	Lower confidence interval	Upper confidence interval	*P*-value^**c**^
^**qSmIA08**^ **AA** ^**qSmIA02/B02**^ **AA** ^**- qSmIA08**^ **BB** ^**qSmIA02/B02**^ **AA**	0.09	−0.01	0.19	0.08
^**qSmIA08**^ **AA** ^**qSmIA02/B02**^ **AA** ^**- qSmIA08**^ **AA** ^**qSmIA02/B02**^ **BB**	0.05	−0.04	0.14	0.51
^**qSmIA08**^ **AA** ^**qSmIA02/B02**^ **AA** ^**- qSmIA08**^ **BB** ^**qSmIA02/B02**^ **BB**	0.19	0.11	0.28	0.00
^**qSmIA08**^ **AA** ^**qSmIA02/B02**^ **BB** ^**- qSmIA08**^ **BB** ^**qSmIA02/B02**^ **AA**	−0.04	−0.14	0.05	0.64
^**qSmIA08**^ **BB** ^**qSmIA02/B02**^ **AA** ^**- qSmIA08**^ **BB** ^**qSmIA02/B02**^ **BB**	0.10	0.01	0.20	0.02
^**qSmIA08**^ **AA** ^**qSmIA02/B02**^ **BB** ^**- qSmIA08**^ **BB** ^**qSmIA02/B02**^ **BB**	0.15	0.06	0.23	0.00

Results of a Tukey HSD test after ANOVA = Mean Incidence ~ AX-147229403 + AX-147212854. ^c^Pairwise comparisons were performed for the means of smut incidence of 103 RILs at AX-147229403 and AX-147212854 SNP positions. ^b^Differences were computed between the maximum smut incidence of each group indicated by the QTL name and the genotype. ^c^*P*-value of the Tukey HSD test (α = 0.05)

### Candidate gene identification for peanut smut resistance

Both QTLs were found within hotspots of disease resistance and defense response genes on the *A. hypogaea cv.* Tifrunner reference genome (https://peanutbase.org). Within the *qSmIA08*, seven gene models are annotated with a potential function in disease resistance. These include nucleotide-binding site leucine-rich repeat (NBS-LRR) genes, NAM (no apical meristem), ATAF1, ATAF2, and CUC2 (NAC) transcription factors, and three mildew resistance locus (MLO) genes (Additional file 7). Within the *qSmIA02/B02*, 31 genes are annotated in the B genome with a potential function in disease resistance, including six disease resistance protein (TIR-NBS-LRR class), 23 LRR/NB-ARC domain disease resistance protein, and two uncharacterized disease resistance protein genes. The region of *qSmIA02/B02* in the A genome retrieved seven annotated genes, including two LRR and NB-ARC domain disease resistance protein and five disease resistance protein gene models (Additional file 7).

## Discussion

Wild relatives of the cultivated peanut have been shown to be sources of resistance to multiple pests and pathogens [29], including the new soilborne disease, the peanut smut [18]. In a previous work we reported on the successful transference of the resistance present in wild species of the A and K genomes into elite lines of the cultivated peanut [21]. In that report, it was also demonstrated that the resistance is highly heritable and was suggested that it may be controlled by few genomic regions. However, the nature of the resistance and the genetic structure was unknown. Here, we used advanced generations (F_7:9_) of the RIL population reported by de Blas et al. [21], to analyze the genome regions conferring resistance in a tetraploid background. The aim of this work was to provide a frame of knowledge for understanding the genetic structure of smut resistance and for future development of markers to aid in the selection of resistant materials in ongoing peanut breeding programs. By developing a high-density SNPs genetic map and phenotypic data measured on the RIL population, we were able to detect one major QTL and a minor QTL for peanut smut resistance, one on LG A08 and other on LG A02/B02, with markers introgressed from the amphidiploid parent.

### Smut resistance evaluation

The results of phenotype evaluation in this work confirm our previous findings on the high stability of the resistance to smut in different RILs when grown under extremely high inoculum pressure [21]. The effective introgression of the resistance from wild diploid *Arachis* species (2*n* = 2*x* = 20) into a tetraploid context (2*n* = 4*x* = 40) of a susceptible elite line of peanut was evidenced in the high (87%) percentage of RILs having significantly lower incidence values than the cultivated parent. This value was even higher than that reported previously for a smaller number of RILs of the same population evaluated in earlier generations [21]. The high heritability of the resistance evidenced a large genetic component of the trait. This population was suitable for the genetic dissection of the trait, as a prelude to understand the genetic architecture of peanut smut resistance in the advancement of commercial cultivars with resistance to smut disease.

### Genotyping and genetic mapping

The genotyping of the 103 RILs retrieved an equal proportion of polymorphic SNP markers for A and B/K subgenomes, as it was expected from the representation in the 48 K ‘Axiom_Arachis2’ SNP array [27, 30]. The difference in number of species-specific markers observed for the diploid progenitors of the amphidiploid may be interpreted in the context of the degree of similarity that each wild species genome has with each other, and with the cultivated peanut subgenomes. That is, the detection of a low number of species-specific markers (99 specific SNPs of *A. correntina* and 98 of *A. cardenasii*) may be explained by the fact that the diploid A genome species have a high sequence similarity with each other and with the A-subgenome of cultivated peanut [30]. By contrast, the ten times higher number (1139) of species-specific markers detected for *A. batizocoi* (K genome) than those detected for the A-genome wild species may be explained by the lower similarity that the K genome has with other genomes. The K genome, although more similar to the B genome species (and to the B-subgenome of cultivated peanut) than to the A genome species [31], has a lower sequence similarity with the B-subgenome of peanut than that reported for the A genome of wild species with the A-subgenome of peanut. Moreover, although recombination between the B and K genomes was reported [32], the mean number of bivalents observed in interspecific BK hybrids is significantly reduced [33]. In this context, although the B-K recombination rate may be low, more markers from the K genome introduced by recombination into the B genome could be detected as species specific than those introduced by the A genome species. This rationale is also supported by the fact that from a total of 3456 *A. hypogaea*-specific markers, 1268 markers hit on the A-subgenome (with E-value <1e-20) and 2158 hits on the B-subgenome (E-value <1e-20). Most SNP markers were inherited as expected for disomic segregation, but others (2.8%) were observed with genotype patterns compatible with homoeologous recombination and tetrasomic inheritance [27, 34]. The percentage observed in the RIL population is similar to that reported previously for a population of similar type and size [34]; and its occurrence was explained under the proposal that peanut is a segmental allopolyploid [34]. Another high proportion of markers (mainly on LGs A04, A05, A07, B03 and B04) showed a distorted segregation with a bias toward *A. hypogaea*. This distorted segregation reveals a strong selection against the amphidiploid alleles. Interestingly, we identified 6 clusters of these markers on five different chromosomes suggesting the existence of segregation distortion regions (SDRs). The SDRs have been shown to be present in interspecific or wide crosses in plants [35–37] and are characterized by harboring genetic selection factors for gametophyte competition [38]. All genes or markers closely linked to a gene that causes segregation distortion and segregates in a population (within the SDR) will also tend to exhibit distorted ratios [39, 40], which may significantly affect plant breeding success. Although none of the QTLs detected in this study was within the SDR, additional research is needed in *Arachis* interspecific populations to better define the direction and rate of segregation distortion and the genetic factors acting within the SDRs.

In *Arachis*, as in other plant genetic systems, the molecular mechanism of segregation distortion remains unclear, and markers showing segregation distortion are usually discarded for map construction [41, 42]. However, diverse studies have shown that markers with segregation distortion could increase the density of markers on the map, increase the genome coverage of the map, and help to refine the detection of QTLs [38, 43–45]. These reports showed that distorted markers can be used for QTL mapping with no adverse effect on the results and can be beneficial if used properly, resulting in a comparative higher genetic variance than non-distortion. Moreover, the use of distorted markers in those reports helped to improve the detection of QTLs and did not have a significant effect on the position or effect estimations of the QTL analysis [44]. In this work we increased the density of the map by adding 889 distorted (up to 4:1) or co-located markers to an initial map constructed with non-distorted markers. This addition did not change the size of the LGs or the position of markers and did not affect the QTL detection. The resulting map with 1819 mapped loci spanning a total of 2531.81 cM is within the range of map density and genome coverage of previous maps constructed for other mapping populations of *Arachis* [35, 46–48]. In most cases, the genetic position of the SNPs was concordant with the physical position on the pseudomolecules of *A. hypogaea* cv. Tifrunner genome [2, 28]. The construction of the LGs A02 and B02 represented a challenge as they were very unstable when the whole set of SNP markers was used as input data. Those LGs were improved after sub-setting the SNP data by their assignation to the *A. duranensis* (Aradu.A02) and *A. ipaënsis* (Araip.B02) chromosomes [28]. This approach enhanced the distribution and number of mapped markers. The LG A02/B02 was retained as part of the map as it held two of the SNPs significantly associated (*P* < 0.01) to the trait after simple linear regression. Although the LG A02/B02 can be assigned to the homoeologous group 2, the genome assignation was not possible. First, this short fragment could not be linked to either LG A02 or LG B02 [even when using lower *P*-value (1e-4 to 1e-6) to determine markers belonging to the same linkage group]; second, only one, out of three SNPs, was previously mapped to Arahy.12 [28]; third, the other two SNPs of the LG A02/B02 showed ambiguous hits on the reference sequence of *A. hypogaea* cv. Tifrunner [2] (https://peanutbase.org). The tight association of markers observed between the LGs A03/B03, and A04/B04 could be explained by homoeologous recombination within tetrasomic genome regions, especially at the distal ends of chromosomes A03/B03 [2]. The linkage map was consistent with the *A. hypogaea* pseudomolecules [2] and showed only one large inversion at chromosome A5 as reported previously [25, 49]. The markers were distributed across the genome, as compared with their physical positions in the tetraploid genome, and the coverage was suitable for QTL identification.

### QTL for peanut smut resistance

As the main objective of the present study, the genetic architecture of resistance to peanut smut introduced from diploid wild species into an elite peanut line was dissected in a tetraploid context. Two genome regions controlling peanut smut resistance explained 17.52% (*qSmIA08*) and 9.06% (*qSmIA02/B02*) of the trait variance. Although *qSmIA02/B02* showed a PVE lower than 10%, which is considered a minor effect QTL [50], there was no significant difference in peanut smut resistance when *qSmIA08* was AA at AX-147229403 and *qSmIA02/B02* was BB at AX-147212854, or vice versa. Results evidenced that these QTLs are the main genetic basis of resistance, which was also supported by the additive effect detected for each QTL. The statistical test of mean differences of RILs harboring both QTLs with alleles from the amphidiploid parent revealed that the disease incidence scores decrease by 57% and were stable across the 3 years of evaluation. Phenotypic analysis of the RILs indicated that the resistance to smut was successfully introgressed from the amphidiploid into an elite peanut line. The marker AX-147229403 within *qSmIA08* region was introgressed from one of the wild diploid A genome species. However, since this marker was not species-specific, it was not possible to determine whether the wild chromosome segment was introgressed from *A. correntina* or *A. cardenasii*. The marker AX-147212854 within the *qSmIA02/B02* interval was also introgressed from one of the wild species used to develop the amphidiploid, however, the source of this resistance could not be determined. Firstly, because the SNP allele was not genome specific; secondly, because the SNP flanking sequences were highly similar in the three wild species, with either the A or K genome. Lastly, the SNP was assigned to the B genome of *A. ipaënsis* in the first version of the array [27], but to the A genome of *A. duranensis* in the second version of the 48 K ‘Axiom_Arachis2’ SNP array [28]. In addition, the *qSmIA02/B02* is mapped to a region of the genome exhibiting tetrasomic recombination [27]. Clear genetic exchange between A and B genomes at the distal ends of chromosome Arahy.02/Arahy.12 was reported by Bertioli et al. (2019). Thus, homoeologous recombinations may increase the uncertainty for tracing the resistant alleles from the wild species. Additional markers would be needed to undoubtedly define the wild sources of resistance. The results of this study reinforce our previous finding indicating that the resistance to peanut smut in the elite peanut line have been introgressed from the wild diploid species [21].

The precision of the QTL position could be mostly affected by population size and heritability [51], and less affected by marker spacing, except in the case of sparse maps with irregular marker spacing. In our study, although the population size was sub-optimal, the heritability was high. The independence observed between the two QTL effects at the haplotypes and SNP positions increases the confidence in the precision of the detected QTLs.

Although only stable QTLs across the 3 years of evaluation were considered in this study, the region with significant association found on LG B09 (9.35 Mbp - 10.97 Mbp) merits further attention as a block of 457 kb on chromosome Arahy.19 has been reported as a potential region associated with resistance to peanut smut [20]. The existence of diverse sources of genetic resistance is relevant for the development of varieties with more durable resistance to smut.

The physical locations of the QTLs detected in this study are regions especially rich for QTLs associated to different disease resistance. Several QTLs for peanut disease resistance have been identified close to or overlapping with the qSmIA08 interval (1.23 Mbp - 6.04 Mbp) including TSWV and ELS [30, 52], and with the *qSmIA02/B02* interval (3.43–4.29 Mbp) including TSWV, ELS and LLS [30, 52]. The relationship for contribution to other traits of the wild introgressed chromosome segment into the tetraploid context awaits future research.

### Candidate gene identification for peanut smut resistance

Transcript density based on Clevenger et al. [53] indicates that resistance gene models (R-genes) within the *qSmIA08* and *qSmIA02/B02* intervals are expressed in reproductive tissues (except two) of *A. hypogaea* cv. Tifrunner, mainly within peg basipetal and fruit platee 1 phenological periods [53]. This is relevant since it was demonstrated that the infection with *T. frezii* begins during pegging [8], the process in which the gynophore enters the soil. These results support the hypothesis that the R-gene clusters found within the confidence interval of QTLs are major candidates to be involved in resistance to peanut smut.

The mechanisms of resistance to peanut smut are largely unknown. However, the candidate genes identified in this study within the QTLs intervals suggest some pathways that need to be further investigated. It is possible that a single gene per QTL may confer resistance, nonetheless Michelmore [54] proposed that genes controlling disease resistance in plants are frequently clustered in the genome and most of these R genes encode nucleotide-binding site (NBS) and leucine-rich repeat (LRR) domains. In peanut and other crops, resistance hotspots (R-gene clusters) have been reported for multiple disease resistance [25, 55, 56]. Here we identified NBS-LRR and TIR-NBS-LRR class genes within the QTLs intervals that are of particular interest for further investigations on smut resistance because they are involved in plant defense responses [57–59].

## Conclusion

In summary, we constructed a genetic linkage map for a novel interspecific RIL population that enabled the genetic dissection of peanut smut resistance trait. One QTL with large effect and another QTL with minor effect, both introgressed from wild diploid *Arachis* species represent different sources of resistance, which is very important for the development of varieties with spatial and time stability of the trait. The fact that both QTLs were located within disease-resistance hotspots containing several R-genes (https://peanutbase.org), and that those genes exhibit high transcription levels in the first steps of fruit development, when the smut infection occurs, constitutes a significant advance in the ongoing research toward the understanding of the mechanisms involved in the resistance to smut. The major and minor effect QTLs detected in this study provide new insights into the genetic architecture of this trait, and a valuable tool for fine-mapping, gene identification, and development of reliable markers for Genomics-Assisted Breeding of new varieties resistant to peanut smut.

## Methods

### Plant material

This study used an advanced generation (F_7:9_) of the 103 RIL population reported by de Blas et al. [21]. They were derived from a cross between a susceptible high-oleic *A. hypogaea* experimental elite line JS17304-7-B (female), hereafter referred to as the cultivated parent, and a synthetic amphidiploid JS1806 (male) hereafter referred to as the amphidiploid [21]. Wild progenitors of the amphidiploid were *A. cardenasii* Krapov. & W.C. Greg (KSSc 36,015), *A. correntina* (Burkart) Krapov. & W.C. Greg (K 11905) and *A. batizocoi* Krapov. & W.C. Greg (K 9484) that belong to the A and K genomes [60, 61].

Seeds of the wild diploid species used in this study were obtained from the Instituto Nacional de Tecnología Agropecuaria, Estación Experimental Agropecuaria (INTA) Manfredi, Córdoba, Argentina. Voucher specimens of the original collections are deposited in the Herbarium of the Instituto de Botánica del Nordeste, Corrientes, Argentina (CTES). All the wild materials were collected between 1958 and 1977 in Argentina and Bolivia before the Convention on Biological Diversity (CBD 1992, https://treaties.un.org), and deposited in National and International seed banks. Seeds of the *A. hypogaea* experimental elite line JS17304–7-B and amphidiploid JS1806 were provided by Criadero el Carmen S.A (peanut nursery located in General Cabrera, Córdoba, Argentina). All field assays were conducted in accordance with local legislation (Law No. 9164, Decree132/05).

### Smut resistance evaluation

Smut resistance was evaluated as previously described [21]. Briefly, the three diploid species used for the development of the amphidiploid, were evaluated in permanent plots, while the tetraploid parents (amphidiploid and cultivated) and the 103 RILs were planted annually. Single-row, 2.5 m long (25 plants) field plots were arranged in a randomized complete block design with three replications, and planted in a *T. frezii* infested soil containing an average of 1.6 × 10^4^ teliospores g^− 1^ of soil (3.5 times higher than the highest value recorded in the cultivated area). The inoculum applied to the soil was a pool of teliospores collected from the whole peanut production area as described in de Blas et al. [21]. All experiments were conducted at Criadero El Carmen nursery located in General Cabrera, Córdoba, Argentina (32°49′40.8″S 63°52′14.0″W) from 2016 to 2018. Pod damage caused by smut was scored on a sample of 100 pods per plot. Smut incidence (Incidence) was calculated as the ratio of affected pods to the total number of pods in the sample [21].

### Statistical analysis

The normality test of phenotypic data was done using the Shapiro-Wilk’s method. To test the global differences of incidence among 103 RILs and controls for each year, a non-parametric Kruskal-Wallis one-way analysis of variance was used at a 5% level of significance (*P* < 0.05) using the “Stats” R package [62]. A full phenotype data set for the 103 RILs was analyzed for incidence using a general linear model on the scale suggested by the Box–Cox transformation to a normal distribution of error terms (square root). The general linear model included genotype, year, and genotype × year as fixed effects. Variance component analysis was done by fitting a linear mixed model from the lme4 R package [63]. Transformed incidence mean values were compared using the Scott and Knott procedure (*α* = 0.05) [64] as implemented in “ScottKnott” R package [65]. The broad sense heritability was estimated according to the formula:

$$ {H}^2=\frac{\sigma_g^2}{\sigma_g^2+\raisebox{1ex}{${\sigma}_{g\times e}^2$}\!\left/ \!\raisebox{-1ex}{$n$}\right.+\raisebox{1ex}{${\sigma}_e^2$}\!\left/ \!\raisebox{-1ex}{$n\times r$}\right.} $$

where ${\sigma}_g^2$ is the genetic variance component among the RILs, ${\sigma}_{g\times e}^2$ is the RIL × environment interaction variance component, and ${\sigma}_e^2$ is the residual component, *n* is the number of years and *r* is the number of replications [66].

A priori analysis for association between disease resistance and SNP markers was performed by running simple linear regression with SNP markers as independent variable and Incidence as dependent variable. Those SNPs with R^2^ *P* < 0.05 were considered as significantly associated to the trait. A one-way ANOVA with a post-hoc TukeyHSD test and Bonferroni’s correction (*P* = 0.05) was carried out to test statistically significant differences of peanut smut incidence among groups of genotypes at the SNP markers positions at the peak of detected QTLs.

### Genotyping

Total genomic DNA was extracted from young leaves and seeds of all the genotypes using the DNeasy PowerPlant Pro Kit (Qiagen, Germantown, MD) according to manufacturer instructions. DNA was quantified with DeNovix DS-11 FX+ Spectrophotometer/Fluorometer (DeNovix Inc., Wilmington, DE) and samples were genotyped with the 48 K ‘Axiom_Arachis2’ SNP array [28]. The genotypic data were processed and analyzed using the Axiom Analysis Suite 5.0.1.38 software (https://www.thermofisher.com). To analyze and filter the output custom Unix scripts were used (Additional file 8). First, genotyping assay results were extracted from SNP calling using a panel of diploid species and the amphidiploid. This set of markers was filtered to reveal SNP markers specific to each of the three parental species and the amphidiploid as follows:

•*A. correntina*/amphidiploid-specific markers: *A. correntina* = amphidiploid ≠ (*A. cardenasii* = *A. batizocoi*).

•*A. cardenasii*/amphidiploid-specific markers: *A. cardenasii* = amphidiploid ≠ (*A. correntina* = *A. batizocoi*).

•*A. batizocoi*/amphidiploid-specific markers: *A. batizocoi* = amphidiploid ≠ (*A. correntina* = *A. cardenasii*).

Second, the three sets of informative SNP markers identified in the first step were matched against a panel constructed with the tetraploid genotypes (cultivated parent, amphidiploid and 103 RIL population) and only those markers that were distinct between the amphidiploid and cultivated parent were kept. The cultivated parent-specific markers (*A. hypogaea*) were also recorded. All SNPs that passed the filter steps were considered for further analyses whether they were mapped or not to *A. hypogaea* cv. Tifrunner genome version [2, 28].

### Genetic map construction

Construction of a linkage map for the RIL population was carried out using “ASMap” R package version 2.0–0 [67]. SNP markers and individuals were filtered out if they contained more than 10% missing data using custom R scripts. Marker deviation from the expected 1:1 segregation ratio was determined by a chi-square test, coupled with Bonferroni’s correction for multiple testing. All markers with the expected segregation ratio (1:1, *P <* 0.05) and minimum percentage of missing data (10%) were selected as frame markers. Those frame markers were considered as highly reliable for the construction of the map. Any co-located marker or with segregation distortion (*P* < 0.05) was excluded from the first map construction, but they were further forced back into the linkage groups constructed with the initial frame markers to densify the map with more SNPs. As recommended by the map construction pipeline of “ASMap”, a simple thresholding mechanism to determine whether markers belong to the same linkage group was calculated using a *P*-value of 1e-6 and a map distance < 35 cM between adjacent markers [67]. The Kosambi function was used to estimate the genetic distances in cM [68]. Given the difficulty in constructing the LGs of chromosome 2, only SNPs mapped to chromosome 2 in the diploids and tetraploid reference genomes [2, 25] were used. Linkage MapView 2.1.2 R package was used to visualize the genetic map [69]. The concordance between the *A. hypogaea* cv. Tifrunner reference genome (https://peanutbase.org) and the linkage map was assessed by comparing the genetic distance (cM) and physical position (Mbp) of each SNP marker, using the R package MareyMap [70]. The physical position of each SNP marker was defined by BLASTn search against the reference genome of *A. hypogaea* [2] (https://peanutbase.org). Pairwise logarithm of the odds (LOD) scores and recombination fractions were calculated to visualize the genetic map using the *heatMap* function.

### QTL analysis

QTL mapping and estimation of additive effects were performed with the R/qtl R package [71, 72]. Allele codes derived from the SNP data of the RILs and progenitors were recorded as follows: homozygous as in the amphidiploid = A, and homozygous as in the cultivated parent = B. Mean phenotypic data (Incidence) of each line was overall and per year estimated and used for the QTL analysis. Conditional genotype probabilities, given the observed marker data, was computed with an error of probability = 0.001. A 1.0 cM window size was used for the genome scan after pseudo-markers function was performed. A Haley-Knott (H-K) regression was scanned to assess the association of each genome position with the trait of interest. The threshold LOD score was estimated empirically for each chromosome using 1000 permutations (*α* = 0.01), and a QTL was declared if the LOD score was over the empirical threshold [73]. The 95% Bayesian confidence intervals were calculated with the *bayesint* function and LOD support interval with *lodint* functions, while the percentage of phenotypic variance explained (PVE) by each QTL was computed with the function *fitqtl* as implemented in R/qtl [71] by fitting a single linear model with each detected QTL and the percentage of reduction of smut incidence was calculated by the differences in percentage of incidence between genotypic classes at QTL positions. In addition, two-QTL scans were performed to assess loci interactions using function *addint*. QTLs were designated following conventional nomenclature with the initial letter *q* followed by the trait name (that was named *SmI* for Smut Incidence), and LG.

### Identification of candidate genes within the QTL intervals

QTL intervals were defined between physical positions of the markers identified at the ends of the confidence intervals using the *lod_int* function in R/qtl2 package [74]. The physical positions were designated according to the reference sequence of *A. hypogaea* cv. Tifrunner [2] (https://peanutbase.org). In order to identify candidate genes potentially regulating disease resistance against peanut smut, resistance genes (R-genes) were retrieved using the Intermine online platform (https://mines.legumeinfo.org/peanutmine/begin.do). Gene expression for each of the gene models detected within QTLs intervals was assessed based on the *A. hypogaea* cv. Tifrunner reference transcriptome [29] (https://peanutbase.org).

## Supplementary Information

**Additional file 1:** List of 56 distorted markers with up to 4:1 segregation ratio distributed in few discrete clusters on LGs A04, A05, A07, B03 and B04.

**Additional file 2:** List of 236 SNPs statistically significant (*P* = 0.01) resulting of a single marker-phenotype association between disease resistance trait and SNP markers.

**Additional file 3:** Collinearity analyses of all of the linkage groups with genome sequences. The x-axis scales the physical positions of markers based on reference sequences. The y-axis represents the genetic distance of the markers in centimorgans accordingly.

**Additional file 4:** Plot of estimated recombination fractions (above diagonal) and LOD scores for tests of *r* = 1/2 (below diagonal) for all pairs of markers in the linkage map. Yellow indicates linkage, while blue indicates pairs that are not linked.

**Additional file 5:** QTLs detected by year for resistance to peanut smut on a RILs population of *Arachis* based on a Halley-Knott genome scan QTL detection model. Resistance to peanut smut is estimated for the level of square root transformed smut incidence; ^a^Year of field evaluation of the trait. ^b^Linkage Group. ^c^Map position in Kosambi cM for each LG. ^d^LOD score at QTL peak.

**Additional file 6:** LOD scores detected on chromosomes A08 and B02 after QTL detection analysis per year. Orange line: 2015, green line: 2016 and purple line: 2017. Red dashed horizontal line indicates the empirical LOD score threshold at *P*-value < 0.05.

**Additional file 7:** Analysis of gene content within the physical intervals of the two QTLs qSmIA08 (1.23 Mbp - 6.04 Mbp) and qSmIA02/B02 (3.43–4.29 Mbp), based on cv. Tifrunner transcriptome information [27] (https://peanutbase.org). ^a^Identifier of a gene from the GeneDB database; bDescription of gene family and protein function identifier when available at www.ebi.ac.uk/interpro/;^Ca^*rachis hypogaea* chromosome ID; ^d^A dot indicates gene expression in reproductive tissue; ^e^A dot indicates gene expression in phenological period between peg basipetal and fruit platee1.

**Additional file 8:** Custom UNIX script for filtering the genotyping data generated in this study.

## Data Availability

The datasets generated and/or analyzed during this study are included in this published article (and its supplementary information files) or available through the Ag Data Commons (National Agricultural Library, USDA Agricultural Research Service): 10.15482/USDA.ADC/1522387.

## Abbreviations

cM: Centimorgan
LOD: Logarithm of the odds
Mbp: Megabase pair
QTLs: Quantitative trait loci
RIL: Recombinant inbred line
SNP: Single nucleotide polymorphism
MAF: Minor allele frequency
