# Supplementary figures and images for: Genetic mapping and QTL analysis for peanut smut resistance
Source: BMC Plant Biol. 2021 Jul 2;21:312. doi: 10.1186/s12870-021-03023-4 (PMC8252251; doi:10.1186/s12870-021-03023-4)

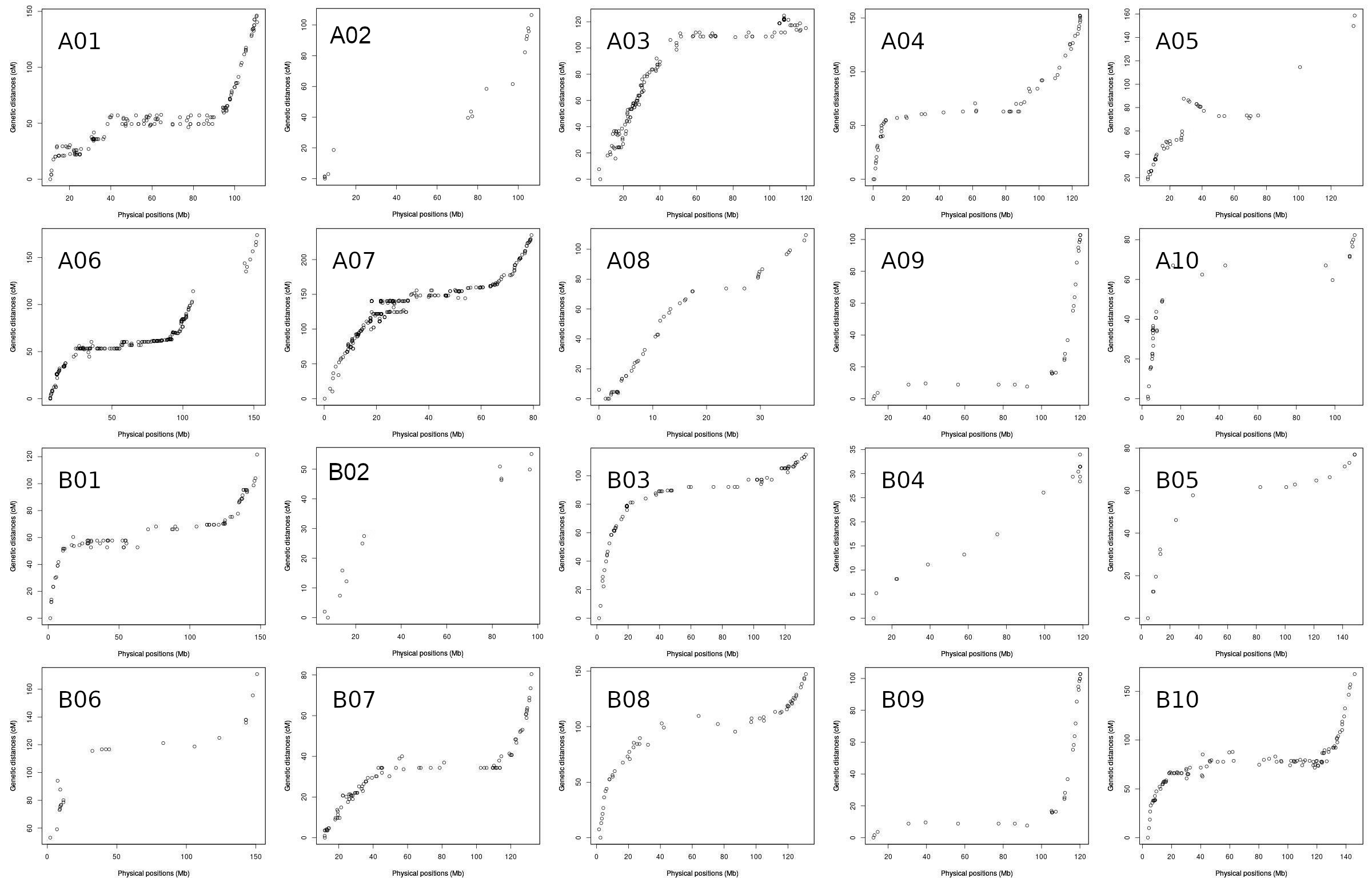

Supplement: Supplementary file 3 — Additional file 3: Collinearity analyses of all of the linkage groups with genome sequences. The x-axis scales the physical positions of markers based on reference sequences. The y-axis represents the genetic distance of the markers in centimorgans accordingly. [file 12870_2021_3023_MOESM3_ESM.png]

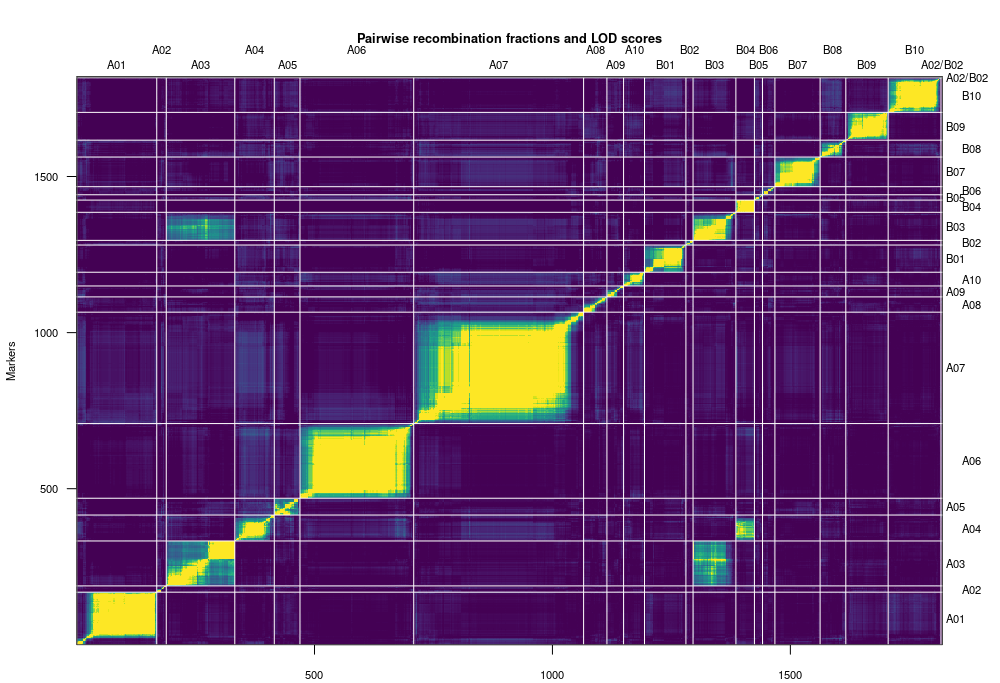

Supplement: Supplementary file 4 — Additional file 4: Plot of estimated recombination fractions (above diagonal) and LOD scores for tests of r = 1/2 (below diagonal) for all pairs of markers in the linkage map. Yellow indicates linkage, while blue indicates pairs that are not linked. [file 12870_2021_3023_MOESM4_ESM.png]

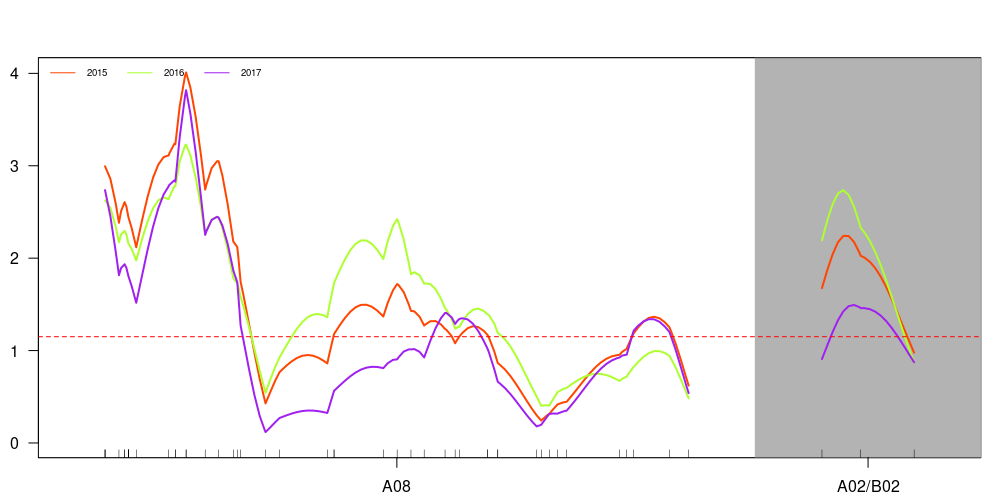

Supplement: Supplementary file 6 — Additional file 6: LOD scores detected on chromosomes A08 and B02 after QTL detection analysis per year. Orange line: 2015, green line: 2016 and purple line: 2017. Red dashed horizontal line indicates the empirical LOD score threshold at P-value < 0.05. [file 12870_2021_3023_MOESM6_ESM.png]
